# Homocysteine, Iron and Cardiovascular Disease: A Hypothesis

**DOI:** 10.3390/nu7021108

**Published:** 2015-02-06

**Authors:** Joseph E. Baggott, Tsunenobu Tamura

**Affiliations:** Department of Nutrition Sciences, University of Alabama at Birmingham, Birmingham, AL 35294, USA; E-Mail: marilynbaggott@att.net

**Keywords:** iron, homocysteine, cardiovascular disease, methionine, thioether

## Abstract

Elevated circulating total homocysteine (tHcy) concentrations (hyperhomocysteinemia) have been regarded as an independent risk factor for cardiovascular disease (CVD). However, several large clinical trials to correct hyperhomocysteinemia using B-vitamin supplements (particularly folic acid) have largely failed to reduce the risk of CVD. There is no doubt that a large segment of patients with CVD have hyperhomocysteinemia; therefore, it is reasonable to postulate that circulating tHcy concentrations are in part a surrogate marker for another, yet-to-be-identified risk factor(s) for CVD. We found that iron catalyzes the formation of Hcy from methionine, *S*-adenosylhomocysteine and cystathionine. Based on these findings, we propose that an elevated amount of non-protein-bound iron (free Fe) increases circulating tHcy. Free Fe catalyzes the formation of oxygen free radicals, and oxidized low-density lipoprotein is a well-established risk factor for vascular damage. In this review, we discuss our findings on iron-catalyzed formation of Hcy from thioethers as well as recent findings by other investigators on this issue. Collectively, these support our hypothesis that circulating tHcy is in part a surrogate marker for free Fe, which is one of the independent risk factors for CVD.

## 1. Introduction

Homocysteine (Hcy) is exclusively formed from methionine (Met) through the transmethylation pathway [1]. In the 1960s, McCully [2] reported that atherosclerosis and thrombosis are common clinical features among patients with homocystinuria (congenital cystathionine β-synthase deficiency) with extremely high circulating total Hcy (tHcy) concentrations (often >500 μmol/L) and suggested that it is detrimental to the integrity of the vascular wall. However, this association received little attention until 1991, when Clarke *et al.* [3] reported that hyperhomocysteinemia is an independent risk factor of cardiovascular disease (CVD); thus, the “Hcy-CVD” hypothesis was born. Since then, Hcy has been subjected to numerous intense investigations focusing on its relation to CVD, and Hcy was a hot topic in nutrition [4]. Intervention trials were conducted to lower tHcy concentration by supplementing B-vitamins (particularly folic acid, pteroylglutamic acid) in order to reduce the CVD risk. However, these trials largely failed to identify a positive effect from the supplementation on the CVD risk despite the reduction in circulating tHcy [5]. In addition, no solid biochemical mechanisms have emerged linking Hcy and vascular damage. These findings indicated that simply lowering tHcy is not effective in reducing the CVD-risk; hence, circulating tHcy may be a surrogate marker of another, yet-to-be-identified, risk factor(s) for CVD. In this review, we summarize results in the literature together with our investigations, which possibly indicate that circulating tHcy is in part a surrogate for non-protein-bound iron (free iron), which is considered to be one of the independent risk factors for CVD [6]. We have searched the literature for articles including tHcy, iron, and CVD; however, in the vast majority of them, the authors did not try to correlate tHcy with iron.

## 2. “Iron-CVD” Hypothesis

Elevated low-density lipoprotein cholesterol (LDL-C) is associated with CVD, and oxidative damage of LDL-C increases its atherogenecity. Oxidatively-modified LDL-C alters the structure and function of endothelial cells and chemotactically attracts monocytes to the subendothelium, where these cells develop into lipid-containing foam cells of atherosclerosis plaque [6]. Iron catalyzes the formation of oxygen free radicals, especially when chelated by EDTA [7], and Sullivan [8] postulated that excess iron leads to atherosclerosis through oxygen free radical oxidation of LDL-C. This hypothesis is controversial, although it is supported by epidemiological data [9]. However, there is evidence that severe iron deficiency is also associated with increased risk of CVD [10], and that both extreme conditions of iron overload and deficiency are associated with increased risk of CVD by different mechanisms, as reviewed by Lapice *et al.* [11].

Major internal exchange of iron takes place through the release of iron from transferrin, ferritin and digested senescent erythrocytes [12]. Uptake of transferrin and release of iron in the acidic endosome occur in nearly all cells, and the amount of iron exchanged is about 0.03 g/day. This endosomal iron is free iron and is capable of catalyzing oxidations [13]. About 50% of hepatic iron exists in ferritin, which is found in the cytoplasm and lysosomes [12]. Lysosomal digestion of ferritin in an acidic environment produces a considerable portion of free iron. About 0.04 g of iron is exchanged daily when senescent erythrocytes are digested in the lysosomes of the reticuloendotherial system. This process recycles the iron to newly formed erythrocytes in the bone marrow [14]. We believe that Met (or Met as a part of protein), *S*-adenosylhomocysteine and cystathionine are constantly exposed to free iron at acidic pHs during iron exchange, leading to Hcy formation.

## 3. Iron-Hcy Interaction

There are only a limited number of human studies on the association between Hcy and iron. Daher and Van Lente [15] reported a significant positive correlation between plasma Hcy and serum iron concentrations in 76 female subjects. Zheng *et al.* [16] also reported higher plasma tHcy in healthy humans given iron sucrose with a Met load than in those given a Met load alone, although the difference was not significant. However, in a study by Facchini and Saylor [17], an average plasma tHcy of 9.0 μmol/L was not altered after iron depletion in patients with type-2 diabetes and glucose intolerance. There is indirect evidence suggesting a link between iron stores and plasma tHcy. For example, plasma tHcy increases with aging, and a similar age-dependent increase in body iron stores are observed as well. Furthermore, plasma tHcy is generally higher in males than females in the adult life, and a similar gender difference is found for body iron stores [4]. We summarize the findings in four groups of research topics in which the iron-Hcy interactions were investigated.

### 3.1. Plasma tHcy and Anticoagulants Used for Blood Collection

The use of EDTA as an anticoagulant raises the possibility that EDTA-chelated iron supports redox cycling in contrast to citrate and other anticoagulants. As summarized in Table 1, Stabler *et al.* [18] reported that a 4-h incubation of whole blood at room temperature increased tHcy by 35% and suggested that erythrocytes produce and export Hcy. Ubbink *et al.* [19] later reported that the mean plasma tHcy increased by about 190% in EDTA-containing whole blood stored at room temperature for 24 h, whereas the increase was only 20% using sodium fluoride (NaF)-containing whole blood, and no such increase was observed when whole blood was stored at 4 °C. They also postulated that Hcy was produced in erythrocytes and exported. Similarly, Andersson *et al.* [20] reported that erythrocytes were the source of the increase in plasma tHcy, when whole blood contained EDTA. They tested the addition of methionine to EDTA-containing whole blood and found only a slight increase in plasma tHcy compared to EDTA alone. These findings in the early 1990s suggest that falsely high tHcy concentrations are found when EDTA-containing whole blood is left at room temperature.

In the late 1990s–2000s, investigators estimated the effect of anticoagulants on plasma tHcy. Willems *et al.* [21] found that citrate-, NaF- and EDTA-containing whole blood resulted in a time-dependent increase in plasma tHcy. However, the increase was significantly lower in whole blood with citrate or NaF than with EDTA. Hughes *et al.* [22] described that the addition of NaF or lithium fluoride (LiF) to EDTA-containing whole blood significantly reduced the increase in plasma tHcy after storage for 4 h at room temperature. They postulated that NaF or LiF reduced the amount of ATP, which would be required for Hcy production. Salazar *et al.* [23] also reported that EDTA-containing whole blood at 0 °C for 6 h showed a greater increase in plasma tHcy than citrate-containing whole blood stored at room temperature, and these results seemingly contradict with those obtained at 4 °C reported by Ubbink *et al.* [19]. Palmer-Toy *et al.* [24] reported that EDTA-containing whole blood at room temperature showed higher plasma tHcy than citrate- or NaF-containing whole blood within 30 min. In contrast, Stabler *et al.* [18] reported that serum (no EDTA) tHcy did not increase in 1 h (room temperature) after blood drawing. It is, therefore, difficult to explain these data simply through Hcy production in erythrocytes.

**Table 1:** Change in plasma total homocysteine (Hcy) concentrations by anticoagulant, temperature and time elapsed before sample processing ^a^.

Researchers	Anticoagulant	Incubation (h)/temperature	Increase in tHcy from Baseline (%)
Stabler *et al.* (1987) [18]	None	1/RT ^b^	0
	None	4; 24/RT	35; 75
Ubbink *et al.* (1992) [19]	NaF ^c^	24/25 °C; 4 °C	20; 12
	EDTA	24/25 °C; 4 °C	188; 15
Andersson *et al.* (1992) [20]	EDTA	24/22 °C	176
	EDTA	4/37 °C	134
Willems *et al.* (1998) [21]	Citrate	4/RT	3
	NaF	4/RT	14
	EDTA	4/RT	26
Hughes *et al.* (1998) [22]	EDTA	4/25 °C	24
	EDTA + NaF	4/25 °C	13
	EDTA + LiF ^d^	4/25 °C	10
Calişkan *et al.* (2001) [25]	Citrate	3/0 °C; RT	3; 14
	NaF	3/0 °C; RT	−5; 12
	EDTA	3/0 °C; RT	3; 18
Duarte *et al.* (2002) [26]	NaF	1; 4/RT	8; 24
	EDTA	1; 4/RT	9; 27
Scheidhauser *et al.* (2005) [27]	NaF + heparin	144/RT	0
	EDTA	168/RT	280
Hübner *et al.* (2007) [28]	Citrate	24/RT	0; 21
	EDTA	24/4 °C; RT	0; 85

^a^ The experiments listed here only those where the actual tHcy concentrations are available and the comparisons between anticoagulants, temperatures and durations elapsed before sample processing (plasma or serum separation). The values were estimated from the graphs when actual values were unavailable; ^b^ Room temperature; ^c^ Sodium fluoride; ^d^ Lithium fluoride.

Calişkan *et al.* [25] found increases in plasma tHcy of 2.4, 1.6 and 1.3 μmol/L in EDTA-, citrate- and NaF-containing whole blood, respectively, which were stored for 3 h at room temperature. Duarte *et al.* [26] reported that plasma tHcy was significantly higher in EDTA-containing whole blood compared to NaF-containing whole blood, where plasma was isolated from whole blood immediately after blood drawing. Scheidhause *et al.* [27] found a similar increase in plasma tHcy from EDTA-containing whole blood which was processed within 15 min after blood drawing. Hübner *et al.* [28] further reported much larger time-dependent increases in plasma tHcy obtained from EDTA-containing whole blood than those in citrate-containing whole blood, although plasma *S*-adenosylhomocysteine concentrations were similar. This is unexpected since *S*-adenosylhomocysteine should be lower from EDTA-containing whole blood, because *S*-adenosylhomocysteine is the precursor of Hcy [1]. In this report, patients with kidney disease had only 0.1–9.5 μmol/L increases in plasma tHcy from EDTA-containing whole blood after storing for 24 h at room temperature. These increases were significantly lower than those of 8.7–23.6 μmol/L found in healthy subjects [28]. These serendipitous findings might be explained by the fact that patients with kidney disease generally have low body iron stores [29], and led us to postulate that iron-catalyzed production of Hcy could be one of the reasons for increases in plasma tHcy from EDTA-containing whole blood.

Collectively, these findings cast doubt on whether Hcy production in erythrocytes and its export were the sole reason for the increase in plasma tHcy from EDTA-containing whole blood. The addition of an inhibitor of *S*-adenosylhomocysteine hydrolase (3-deazaadenosine) to EDTA-containing whole blood did not eliminate the time-dependent increases in plasma tHcy [30,31]. *S*-adenosylhomocysteine hydrolase activity would be necessary for Hcy production in erythrocytes [1]. As stated above, iron chelated by EDTA is active in redox cycling, whereas much less so when it is chelated by citrate or is not chelated (e.g., NaF) [7]. Therefore, we suspected that the presence of EDTA is part of the reason for increased plasma tHcy. As shown in Table 1, there is a wide variation in the changes in tHcy concentrations between studies, probably due to the heterogeneity in experimental conditions. Furthermore, it can be concluded that the effect of EDTA on the increase in tHcy is time- and temperature-dependent. These data led us to study the *in vitro* effect of free iron on Hcy production from Met and other thioethers [32].

### 3.2. In Vitro Formation of Hcy from Met, S-adenosylhomocysteine and Cystathionine

**Figure 1:**
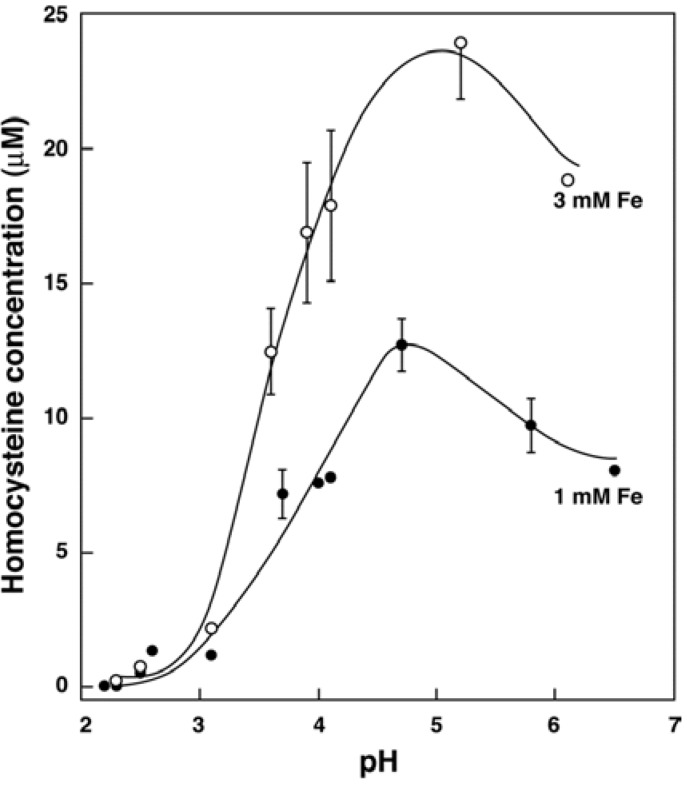
pH profiles for the formation of homocysteine from methionine. Methionine (5.0 mM) in 5.0 mM citrate buffer was incubated with two concentrations of FeSO_4_ (2 h at 37 °C). Error bars represent mean deviations. When iron was eliminated from the reaction, homocysteine concentrations were below the detection limit (<0.05 μM). Adapted with permission from Baggott, J.E. and Tamura, T. [32], Nature Science Grouping © 2007.

We tested whether Hcy is formed from Met *in vitro* in the presence of free iron at various pHs [32]. Figure 1 shows the iron and pH dependence of the reaction. The higher iron concentration resulted in greater Hcy production, and the peak was observed at pH 4.5–5.5. When 1-[^14^C]-Met was used as a substrate, [^14^C]-Hcy was formed. Peaks of [^14^C]-Hcy and fluorescence-derivatized Hcy were coeluted from the HPLC column, indicating that Hcy formed from Met was not an artifact [32]. The addition of ascorbate (1.0 mM) increased Hcy formation from Met (1.0 mM FeSO_4_ at pH 5.5) by 23% in citrate buffer and by 103% in EDTA buffer. These results would be expected if redox cycling of iron to produce reactive-oxygen species [7] is important in the demethylation of Met. These findings suggested that Met could be demethylated to Hcy under acidic conditions *in vivo*, such as in the stomach where the hydroxyl radical is formed [33]. Similar experiments were performed using *S*-adenosylhomocysteine and cystathionine. Both produced Hcy, and the absolute requirement of iron and a similar pH dependency were found [32]. The concentration of iron used in our experiments was physiologically plausible because the concentration of iron in lysozomes of the kidney is as high as 1.7 mM in humans [34]. This set of experiments indicated that there is an iron-dependent formation of Hcy from thioethers *in vitro* and led us to conduct the following human study [32].

### 3.3. In Vivo Changes in tHcy after a Met Load with/without Iron

**Figure 2:**
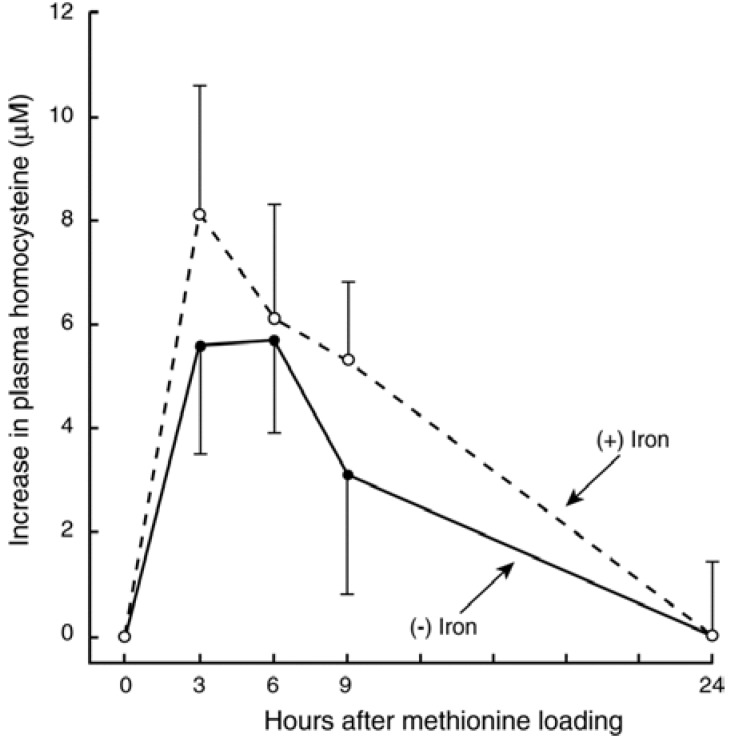
Changes in plasma total homocysteine concentrations after methionine load with or without FeSO_4_ in five subjects. Mean (±SD) concentrations over baseline are plotted. Dashed and solid lines represent Met (0.17 mmol/kg of body weight) load with and without FeSO_4_ (13.0 μmol/kg of body weight), respectively. The mean AUC after methionine load with iron was significantly greater than that after methionine alone (*p* < 0.025, paired-Student’s *t* test). Adapted with permission from Baggott, J.E. and Tamura, T. [32], Nature Science Grouping © 2007.

To evaluate whether an iron-dependent chemical reaction converting Met to Hcy occurs *in vivo*, we performed oral Met-load tests with and without co-administration of iron in healthy adults. Met (25 mg/kg body weight) was given orally with or without iron (FeSO_4_ 3.74 mg/kg body weight). Blood samples were obtained before and after the doses (Figure 2). The mean area-under-the curve after Met with iron (89.7 μM × hour) was 44% higher than that after the load of Met alone (62.1 μM × hour, *p* < 0.025). Our data clearly suggest an iron-dependent conversion of Met to Hcy *in vivo* in humans [32].

### 3.4. Recent Studies on Iron and Hcy in Humans

In this section, we summarize recent reports suggesting that iron is related to circulating tHcy in humans, although these are not direct support to our hypothesis. Mathioli *et al.* [35] found a significant correlation between serum iron and plasma tHcy in male patients with myocardial infarction (*r* = 0.83) and healthy controls (*r* = 0.34). LDL-C was also significantly correlated with serum iron in these patients (*r* = 0.88). Schiepers *et al.* [36] conducted a study of 818 patients with plasma tHcy of 13–26 μmol/L, who were enrolled in a prospective trial. In response to our letter [37], Schiepers and Durga [38] reported a significant positive correlation between plasma tHcy and serum ferritin (*r* = 0.125); however, they did not believe that there was a cause-effect relationship. Lioupis *et al.* [39] found a significant independent positive relationship between quartiles of plasma tHcy with iron content in the carotid-artery plaque in patients undergoing carotid endarterectomy. They suggested that elevated plasma tHcy could promote the formation of reactive-oxygen species, which is the opposite of reactive-oxygen species causing elevated plasma tHcy. Furthermore, Pena-Duque *et al.* [40] showed a significant positive correlation (*r* = 0.66) between plasma tHcy (range: 5.0–16.0 μmol/L) and aortic-artery iron content in patients undergoing coronary-artery-bypass surgery. They reported that the mean aortic-artery iron content was 8-times higher than that in the mammary artery. Proteins and amino acid in circulation would be constantly exposed to these iron-containing plaques.

Pimkova *et al.* [41] reported that mean plasma tHcy of 14.5 μmol/L in patients with myelodysplastic syndromes was greater than that of 9.4 μmol/L in healthy controls. In these patients, mean plasma ferritin was elevated (although not significantly), whereas plasma iron was normal. Plasma malondialdehyde (an indicator of oxidative stress) concentrations had significantly positive correlations with serum free iron (*r* = 0.60) and log ferritin (*r* = 0.78), suggesting reactive-oxygen species production was related to iron stores.

As a note of caution, with the exception of the study by Schriepers *et al.* [36], the number of patients examined in these investigations was less than 100. Finally, in a review of oxidative stress in Alzheimer’s disease, Greenough *et al.* [42] suggested that plasma tHcy, reactive-oxygen species, oxidative damage and iron stores are associated in this patient population, where plasma tHcy was often elevated. These findings suggest that plasma tHcy is positively associated with circulating iron concentrations or iron deposits in arterial plaques. This relationship, regardless of the validity of the “iron-CVD” hypothesis [8], suggests the existence of an iron-Hcy interaction.

## 4. Conclusions

It is remains to be determined how hyperhomocysteinemia is etiologically related to CVD risk, since the lowering of plasma tHcy concentration did not reduce the risk. Our concern about mildly elevated tHcy being an independent risk factor for CVD is not new. In an extensive review, Brattstrӧm and Wilcken [43] questioned whether Hcy was a cause or effect of CVD, because the homozygous genotype (TT) of 5,10-methylenetetrahydrofolate reductase C677T polymorphism is not associated with increased risk of CVD, although it leads to elevated tHcy. They also raised the possibility that anti-oxidant therapy reverses Hcy-induced oxidative damage on the vascular system. However, at present, there is no solid mechanism to link tHcy and CVD. We propose that mildly elevated plasma tHcy *per se* may not be as detrimental to health as many investigators believe. In contrast to hyperhomocysteinemia, the relationship of iron to CVD is scientifically much tighter in terms of chemical and biochemical mechanistic links. Based on our results and the studies reviewed above, we hypothesize that an iron-dependent chemical reaction, which converts thioethers to Hcy, occurs in nearly every cell of the body; therefore, plasma tHcy concentration is, to a certain extent, a surrogate for body iron stores and Met intake. We hope that our view can fill, in part, the gap in the understanding of the mechanistic interaction of Hcy and CVD. The “Hcy-CVD” hypothesis regrettably began with the report showing an odds ratio of 3.2 for CVD in only 123 patients with vascular disease, together with only 27 controls [3]. A monumental amount of basic, clinical and clinical-trial research followed, and it is undeniable that we learned a great deal about Hcy metabolism, physiology and chemistry. However, lack of solid etiological links for the hypothesis suggests that resources committed to this hypothesis were out of proportion compared to other areas of CVD research. Are we the only ones who think that resources could have been used in a more productive way?
